# An Algorithmic Model of Decision Making in the Human Brain

**DOI:** 10.32598/bcn.9.10.395

**Published:** 2019-09-01

**Authors:** Sohrab Saberi Moghadam, Farid Samsami Khodadad, Vahid Khazaeinezhad

**Affiliations:** 1. Faculty of Engineering Modern Technologies, Amol University of Special Modern Technologies, Amol, Iran.; 2. Department of Physiology, Faculty of Biology and Medicine, University of Lausanne, Lausanne, Switzerland.; 3. Department of Computer Engineering, Faculty of Engineering, Ayatollah Amoli Branch, Islamic Azad University, Amol, Iran.

**Keywords:** Decision-making process, Model predictive control, Memory structure, Prefrontal cortex, Hippocampus

## Abstract

**Introduction:**

One of the interesting topics in neuroscience is problem solving and decision-making. In this area, everything gets more complicated when events occur sequentially. One of the practical methods for handling the complexity of brain function is to create an empirical model. Model Predictive Control (MPC) is known as a powerful mathematical-based tool often used in industrial environments. We proposed an MPC and its algorithm as a part of the functionalities of the brain to improve the performance of the decision-making process.

**Methods:**

We used a hybrid methodology whereby combining a powerful nonlinear control system tools and a modular fashion approach in computer science. Our hybrid approach employed the MPC and the Object-Oriented Modeling (OOM) respectively. Therefore, we could model the interaction between most important regions within the brain to simulate the decision-making process.

**Results:**

The employed methodology provided the capability to design an algorithm based on the cognitive functionalities of the PFC and Hippocampus. The developed algorithm applied for modulation of neural circuits between cortex and sub-cortex during a decision making process.

**Conclusion:**

It is well known that the decision-making process results from communication between the prefrontal cortex (working memory) and hippocampus (long-term memory). However, there are other regions of the brain that play essential roles in making decisions, but their exact mechanisms of action still are unknown. In this study, we modeled those mechanisms with MPC. We showed that MPC controls the stream of data between prefrontal cortex and hippocampus in a closed-loop system to correct actions.

## Highlights

- A conceptual framework is designed for modeling decision path according to human cognitive planning.
- A high-level algorithm is developed for data flow in the human brain decision region.
- In this article, the flow of the sensory data analysis is controlled in the brain by Model Predictive Controller (MPC).

## Plain Language Summary

Decision-making is a cognitive process of the human brain. The brain behaves as a complex system, and providing a model would be a convenient way to represent the complexity of the brain. Every decision includes some stages: each stage can be interpreted as a cognitive criterion. The brain controls the path by predicting the action’s result. The brain needs to know the criteria to perform its primary function as a predictor. It is known that the hippocampus stores the knowledge, and the prefrontal cortex approximates the goals; therefore, our study models the interactions between the hippocampus and the prefrontal cortex by providing an algorithmic view. In our model, the effects of the brain regions controlling the path are replaced by the model predictive control. Now the neurological mechanisms of the decision-making process in the brain can be simulated. This capability allows us to Work on some sort of neural networks diseases such as neurodegenerative disease or some rehabilitations, which needs memory consolidation.

## 1. Introduction

Decision-making is a recurrent, ubiquitous cognitive process and a consequential part of human behavior. A decision is made out of a set of items based on specific criteria. It is widely accepted in cognitive science that damage to the frontal lobe in the brain impairs one’s ability to make a decision (Ratcliff & Rouder, 1998). The researchers are still studying the relationship between thinking and automated human activities (Barraclough, Conroy, & Lee, 2004; Hasegawa, 2000; Wang, 2008), including the control of the decision-making process. By putting the prediction horizon and control horizon of a given control system together, we can take a step forward in increasing the trust and precision of decisions (Ramírez & Camacho, 2006). In our study, the intelligent control of decision-making process based on prior experience will improve brain functionality under a new conceptual control role. It results in the promotion of a learning model based on prior knowledge.

In recent years, many efforts have been made to design various decision-making models using different kinds of neural networks such as neural networks in distributed decision making using the Kalman filter series (Gers & Schmidhuber, 2000) and fuzzy models, models which are based on primary knowledge and experience. While the field of decision-making research continues to be vibrant, several researchers, including Dawes (e.g. Hastie and Dawes, 2001), Loewenstein (e.g. Camerer et al., 2005), and Mellers (2000) have proposed that the next phase of research in this area is likely to emerge from building on recent advances in the field of neuroscience. Since 1990, neuroscience methods and decision-making were combined to investigate the nature of decision-making, brain structures, and neurological mechanisms (Niwa & Ditterich, 2008; Romo, Hernández, Zainos, Lemus, & Brody, 2002; Wong & Wang, 2006). The primary objective of this article is to predict the correct decision making path and control of these complex mechanisms, which results from human cognitive planning.

The Prefrontal Cortex (PFC) and hippocampus are the most critical parts of the human brain for decision making. The decision-making process contains four steps. In the first step, some initial stimuli produced by sensory inputs, excite a set of hippocampal neurons as part of the neural system. In the second step, a set of secondary stimuli arrives in the hippocampus, and the stimulusdriven neural response is produced as initial information for two entry stimulus sets in the hippocampus. In the third step, the initial information is sent to PFC. The PFC determines the additional required information and retrieves complementary information from the hippocampus (Wang, 2008). In the last step, the PFC decides the proposed controlling process in this study. However, there is a mutual communication between PFC and hippocampus with neural connectivity. This neural wiring makes closed-loop neural circuits to generate a preferred decision.

## 2. Model Predictive Control

This paper is based on the concept of a Model Predictive Controller (MPC) presented by Enkavi et al. (2017). MPC is used for path planning (trajectory) of autonomous actions and formation control. MPC is a proper technique for optimizing the efficiency of control systems, The MPC is a proper technique for optimizing the trajectory control problem. The MPC Controller tries to minimize the cost in each sample time by taking the initial and current states, and the optimization process calculated from the current state in the next loop.

**Figure 1.**
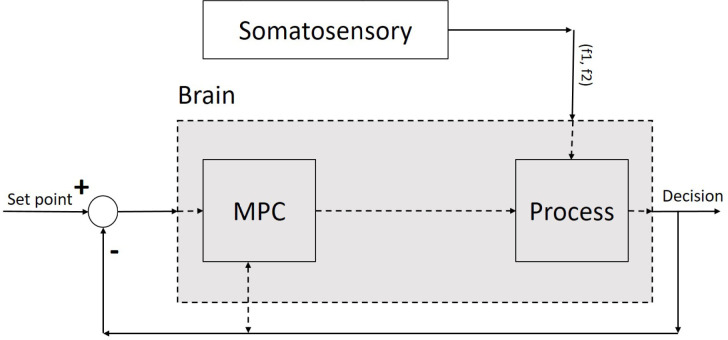
Model predictive control system for a decision-making process

As seen in Figure 1, the MPC (controller) is used to control the process (part of the brain) to make an optimized decision on a sequence of decision items. We proposed the MPC for a class of nonlinear discrete-time systems using the constraint positively invariant sets (Figure 1). We construct the algorithm by two control modes: The state feedback mode for keeping the state (decision items) in a set (decision plan) and the MPC mode for steering the state to the set. A typical cost function in MPC in our nonlinear discrete-time control systems is as follows Formol 1, 2, 3, 4 & 5:

1. $$J=min\sum_{k=0}^{N-1}{\left\|\hat{y}(k+H_{p})-r(k+H_{p})\right\|}^{2}+\rho {\left\|u_{Hp}+k)\right\|}^{2}$$

2. $$u={\left[u(k),u(K+1),\ldots ,u(k+H_{p}-1)\right]}^{T}$$

3. $$\hat{y}={\left[\hat{y}(k+1),\hat{y}(k+2),...\hat{y}(k+H\_p)\right]}^{T}$$

4. $$r={[r\mathrm{(k}+\mathrm{1)},\mathrm{r(k}+\mathrm{2)},\ldots ,\mathrm{r(k}+H_{p})]}^{T}$$

5. $$\mathrm{Constraints}[\begin{array}{l} u_{\min}\leq u_{Hp+k})\leq u_{\max}\\ u_{\min}\leq u_{Hp+k})\leq y_{\max} \end{array}$$,

where u (k), y (k),) and ŷ (k) denote the controller output, the process output, and the predicted process output, respectively at time instant k, r (k) is the desired output or the set points (desired decision items), and H (P) is the predicted horizon step. At time k, MPC solves an optimal control problem over a finite future horizon of H_p_ step and receives a new controller output sequence u(k+1) and repeats the optimization at time k+1 by minimization of J concerning u (k) and soon.

**Figure 2.**
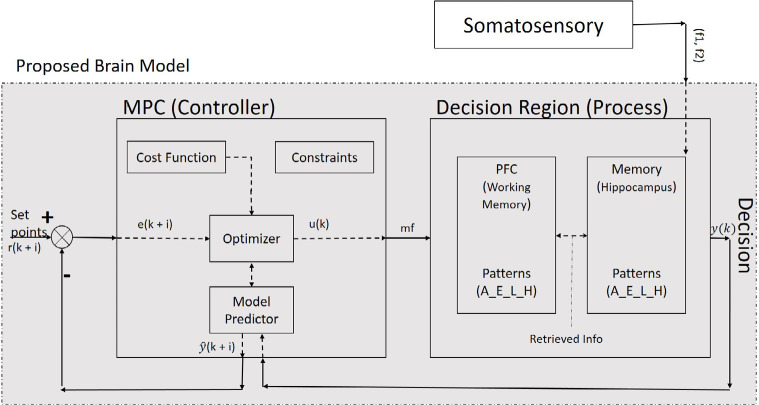
Proposed model for a closed-loop system of the spiking neural network: a decision-maker

The main problem to be solved here is to find a model, which could choose proper decision items in the decision plan and steer it in a state space from its current location (status) to its goal. Status is the understanding of a goal in control iterations (Figure 2). The state space is all possible states in the problem environment.

## 3. The Proposed Model

Since the decision region (Figure 2) is related to the hippocampus and PFC (Weilbächer & Gluth, 2016), our process contains a set of neurons of both PFC and hippocampus. Likewise, the offered model contains a controller and set of the neural network as a process, which is interrelated and has nonlinear and dynamic parts. The model has a long-term memory (hippocampus), which maintains the classified sensory information (Enkavi et al., 2017). It can retrieve the classified information in PFC based on the properties of neurons. The input of the proposed process is sensory stimulation, and its output is a discrete signal, which contains decision item types. Therefore, the offered model with a dynamic property has a structure based on saved knowledge and experience which results in the next decision. In other words, the output of the MPC controller, as a controlling signal input of our process block (Figure 1) and changes in our neural network weights is based on experience analogous to human memory.

**Figure 3.**
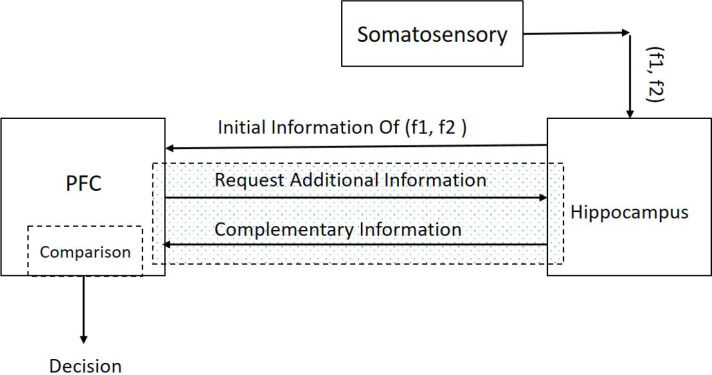
The interaction between PFC and hippocampus for retrieving the required information

Suppose a sequence of decision items (desired set points) like (A-E-L-H) are created and classified based on forming cognitive planning. Figure 2 illustrates that decision-making could result from a comparison between the sensory stimuli of two frequencies (f1 and f2) in the specific area of the brain in the decision region (Haegens, Nácher, Luna, Romo, & Jensen, 2011). In this paper, the decision region is composed of the hippocampus and PFC and neural connectivity’s between them (Figure 3).

While some interactive signals interchange through the main parts of our model, the decision is made. The first part of our model, which takes the initial signal from the somatosensory system is hippocampus. Hippocampus is in pre-exited mode and waits for stimulation from somatosensory area. When f1 reaches the permanent memory in the hippocampus, the neural network transients to the exciting or loading mode (Wang, 2008). After a few seconds, the second sensory stimulus from somatosensory area (f2) reaches to the hippocampus. The first and secondary stimulus (f1 & f2) create a neural response in the hippocampus (Figure 3). The PFC would be aware of the situation by taking this neural response as initial information.

The PFC goes to comparison mode by receiving the initial information. The PFC based on this received information sends a request to hippocampus for additional information. Hippocampus responses with appropriate saved knowledge as complementary information to the working memory in the PFC. This mutual communication (request and response) is called a retrieval mechanism (the hachure region in Figure 3). The retrieved knowledge contains decision-items such (A-E-L-H). These patterns maintained in working memory as a set point. For example, item A is our first target in a decision plan. The decision plan is controlled by the output of the MPC system, as a controlling signal, which is called the Modified Frequency (MF).

The MPC controller works under constraints, and cost function needs to find the best-predicted items closest to the desired set points (decision plan [A-E-L-H]). Therefore, it generates multiple future scenarios in a systematic way where an optimizer comes into the MPC controller block. A copy of the decision-items (output of process) goes to the MPC and is compared to the desired set points. By solving an optimization problem, the MPC controller tries to minimize the difference between the desired set points and predicted items of decision items (A-E-L-H).

**Figure 4.**
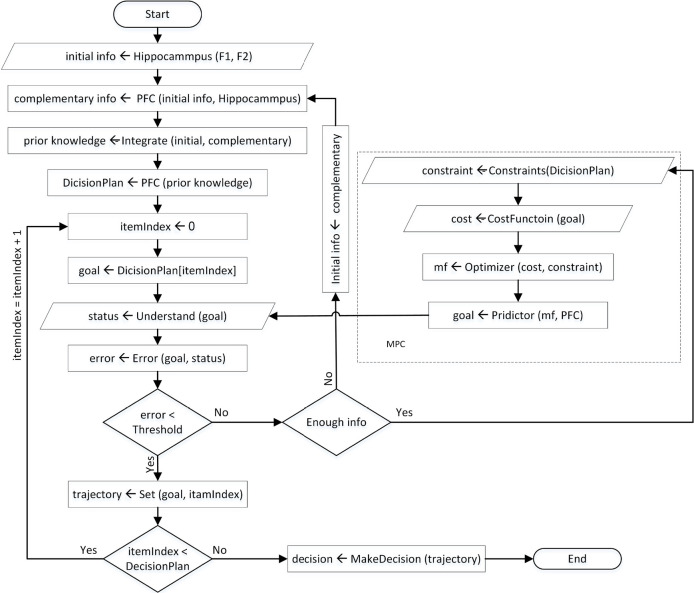
Decision-making flow chart of our proposed model

The output of the MPC block produces the future controlling signal over the prediction horizon and predict the MF, which drives brain response to make a correct decision. The cost function of this optimization problem is represented as a weighted squared sum of the predicted errors (Eq. 1). Moreover, MPC ensures that the MF and correct decision making stays within pre-determined limits (Eq. 5). These are referred to as constraints such as the range of amplitude and frequency of brain responses during the decision process. Generally, the MPC controller is solving the optimization problem over the prediction horizon while satisfying the constraints. The predicted item with the smallest cost function gives the optimal solution and therefore determines the optimal MF (outputs of MPC) that will get the predicted item as close as possible to the desired set points. Figure 4 shows the algorithmic modeling for the process of decision making. This flow chart represents our proposed model systematically.

In other words, a combination of model responses (saved knowledge), past controlling signal (MF), desired set points (decision plan), and process output create the future output process, and finally, the future decision is made. Therefore, in the control process, we can obtain a new desired set points signal in each feedback loop. It means that if we want to go from item (A) to (E), we choose (E) as the desired set points, and we try to reach decision item (E) based on prediction horizon and MPC control horizon. Then in the next closed loop, we choose a new-desired set point (L), and the purpose is to reach a new decision point.

## 4. Discussion

It is believed that damage to the brain frontal lobe may impair one’s ability to think and make decisions. The precise process of the prefrontal cortex is still unknown. In this paper, by offering a universal algorithmic model, we replaced the non-specific area of the brain with an MPC controller to mimic the frontal lobe function. To make a decision, we have assumed four main activities that may be part of the control design, including internal and external properties of neuronal subpopulations. MPC is planning to develop the model with more stability and robustness against disturbances and noise.

The proposed model has the predictive and control horizons to optimize future decision plan with higher accuracy compared with the other methods. Since the human brain has a set of neurons with corresponding complexity, it seems that the decision-making and cognitive process of the brain can solve this complexity in an advanced control block. Therefore, to reach this horizon, we tried to combine both hippocampus and prefrontal cortex parts (decision area) and consider this combination as a model process and take advantage of MPC for this model. In other words, model process is defined with the neural wiring between cortical and subcortical networks in a closed-loop control system. It is worth mentioning that the change of electrical properties of neural responses (frequency and amplitude) create some specific information in decision area. The change of these properties determines a decision signal in a comparison mode.

## Ethical Considerations

### Compliance with ethical guidelines

There is no ethical principle to be considered doing in this research.
